# Supplementary material for: MetaRibo-Seq measures translation in microbiomes
Source: Nat Commun. 2020 Jun 29;11:3268. doi: 10.1038/s41467-020-17081-z (PMC7324362; doi:10.1038/s41467-020-17081-z)
Supplement: Supplementary file 10 — Supplementary Data 7 [file 41467_2020_17081_MOESM10_ESM.zip › File2/Confidence_High_Taxonomy/283299_out.krona.html]

Javascript must be enabled to view this page.

members
magnitude
magnitudeUnassigned
count
unassigned
taxon
rank

283299\_out

3

2
3
superkingdom

phylum
3
1224

3
class
1236

91347
3
order

543
family
3

genus
3
561

species
3

SRS049402\_contig\_number\_contig-100\_810.117532SRS076876\_contig\_number\_160SRS147022\_contig\_number\_11277
562
